# Two-Step Parametrial Endometriosis Nodule Excision Using Virtual Reality Technology and 3D Modelling for Surgical Planning

**DOI:** 10.1155/crog/5513823

**Published:** 2025-05-27

**Authors:** Rooma Sinha, Sukhbir Singh, Teresa Flaxman

**Affiliations:** ^1^Department of Gynaecology and Robotic Surgery, Apollo Hospitals, Hyderabad, Telangana, India; ^2^Macquarie University, Sydney, Australia; ^3^Department of Obstetrics, Gynecology, and Newborn Care, The Ottawa Hospital, Ottawa, Canada; ^4^Department of Obstetrics and Gynecology, University of Ottawa, Ottawa, Canada; ^5^Clinical Epidemiology Program, Ottawa Hospital Research Institute, Ottawa, Canada; ^6^Department of Radiology, Radiation Oncology and Medical Physics, University of Ottawa, Ottawa, Canada

## Abstract

Extensive and infiltrative fibrous adhesions of the uterus and ovaries to the surrounding organs make surgical interventions in endometriosis challenging. A preoperative identification of these involvements can help the surgeon better prepare for the surgery. Traditionally, ultrasonography and magnetic resonance imaging (MRI) have been used. However, clinical use of modern VR technology for creating and visualising a three-dimensional (3D) digital model for a complex surgical case has been proposed. We describe a case of a 29-year-old who presented with dyspareunia and dysmenorrhea (VAS score of 10/10) with left parametrial endometriosis and created a 3D model from their two-dimensional (2D) DICOM images. A left parametrial endometriosis nodule was identified involving the left ureter, rectum, and vaginal fornix along with mucosa. A virtual preoperative surgery was done for precise and complete excision of the disease and to prevent injury to the left ureter and rectum. The surgery was performed as a two-step excision using a da Vinci Xi robot and included left ureterolysis, shaving of the bowel endometriosis nodule and full-thickness vaginal wall excision along with the infiltrating nodule. The infiltrating endometriosis nodule was split into two halves and was excised individually. Her postoperative VAS score for dysmenorrhea was 2/10, and she is 28 weeks pregnant at the time of submission. Advanced VR imaging can help in the evaluation and management of deep endometriosis. It can improve the surgeon's understanding of the specific anatomy, visualise the disease, and improve clinical outcomes.

## 1. Introduction

Endometriosis is a neighborhood disease. It involves organs in the pelvis, and the extent of involvement determines the surgical steps and extent of excision. The focused resection of endometriosis lesions and their extent of clearance are crucial in postoperative clinical outcomes and prevention of recurrence. The excision should not result in bowel, bladder, or ureteric complications. The parametrium is the connective tissue around the cervix and comprises three ligaments (cardinal, sacrouterine, and vesicouterine). The parametria consist of the visceral pelvic ligaments and have the vascular, lymphatic, and neural structures within the two layers of visceral pelvic fascia. In each hemipelvis, the parametrium is the pathway for lymphatics, vessels, and nerve fibres from the pelvic viscera towards the lateral pelvic wall. This parametrium is divided into anterior, lateral, and posterior parametria. Dissection of the pararectal and paravesical spaces can help identify and resect anatomic structures [1].

When we find endometriosis affecting the parametrium, radicality and tailored surgical procedures must be balanced for the excision of the disease and preserving the vascularity and lymphatics to the uterus, along with the pelvic splanchnic nerves. Detailed imaging helps in surgical planning as well as patient counselling. Traditionally, transvaginal ultrasonography (TVS) and magnetic resonance imaging (MRI) have been used extensively, and these have been recommended in most guidelines, including ESHRE in 2022 [2].

While both TVS and MRI are used as imaging modalities for diagnosing endometriosis, TVS is better for detecting ovarian cysts and bladder lesions. MRI is better for diagnosing a deep infiltrating lesion of the uterosacral ligaments, rectovaginal space, or bowel. Both methods are complementary and effective and can be combined for better preoperative endometriosis evaluation [3].

Volumetric medical images can create patient-specific three-dimensional (3D) constructs from two-dimensional (2D) DICOM image sets. These 3D constructs, or 3D models, can be viewed on a computer screen, printed as physical objects using a 3D printer, or visualised using modern virtual reality (VR) technology. 3D models made from medical images are advantageous because a user can isolate structures of interest, customize the appearance with specific colour and transparency, and view the model from any angle. The clinical use of 3D models has been shown to improve the anatomical comprehension of physicians as they can provide a more accurate assessment of anatomical volumes and improve one's understanding of structural relationships [4–6].

We report the use of modern VR technology in a clinical setting for both the creation and visualisation of a 3D digital model for a case of left parametrial endometriosis disease involving the left ureter, rectum, and full thickness of vaginal mucosa.

## 2. Case History

A 29-year-old female presented with dyspareunia and dysmenorrhea. She had mild to moderate dysmenorrhea for the last 5 years, which had increased recently to a VAS score of 10/10. She complained of dyspareunia for the past 6 months since becoming sexually active. Her BMI was 21.55 kg/m^2^. She has regular menstrual cycles with bleeding for 5 days with normal flow. She was nulligravida at the time of the presentation.

**Figure 1:**
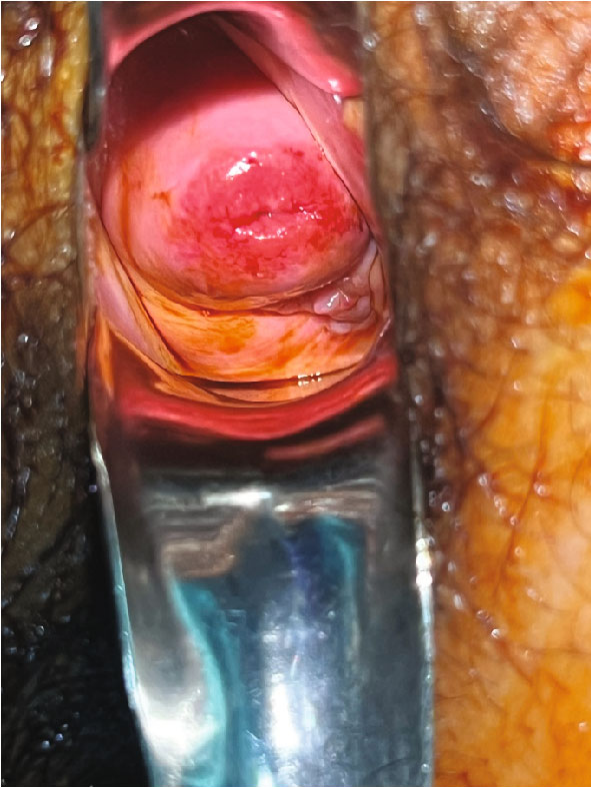
Endometriosis infiltrating the left vaginal fornix.

The general and abdominal examination did not reveal any abnormality. Speculum examination confirmed an irregular mass at the left vaginal fornix of 2 × 2 cm (Figure 1). On palpation, this mass was fixed to the vaginal mucosa and felt firm in consistency. On bimanual examination, the left fornix and uterosacral ligament were firm, with restricted mobility. The posterior fornix had fixed and nodular consistency. Rectal mucosa was free on digital rectal examination, and nodularity was felt 5 cm from the dentate line.

**Figure 2:**
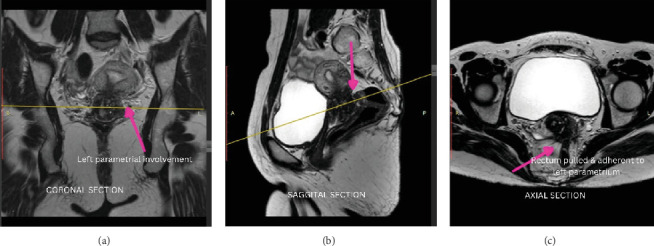
T2-weighted MR images in the (a) coronal, (b) sagittal, and (c) axial planes depicting an endometriosis nodule in the left parametrium involving the rectum and vagina.

MRI was performed, and a well-defined hypodense lesion (21 × 19 mm) was reported anterolateral to the cervix on the left side. This mass extended to the left parametrium. There was a loss of interface with the bladder anteriorly and the rectum posteriorly (Figure 2).

A differential diagnosis of the left parametrial endometriosis nodule with vaginal wall and mucosa involvement or vaginal carcinoma extending to the left parametrium was made. We examined under anaesthesia and diagnostic hysteroscopy. The biopsy from the lesion was reported as endometriosis. She was counselled by robot-assisted excision of the left parametrial endometriosis.

**Figure 3:**
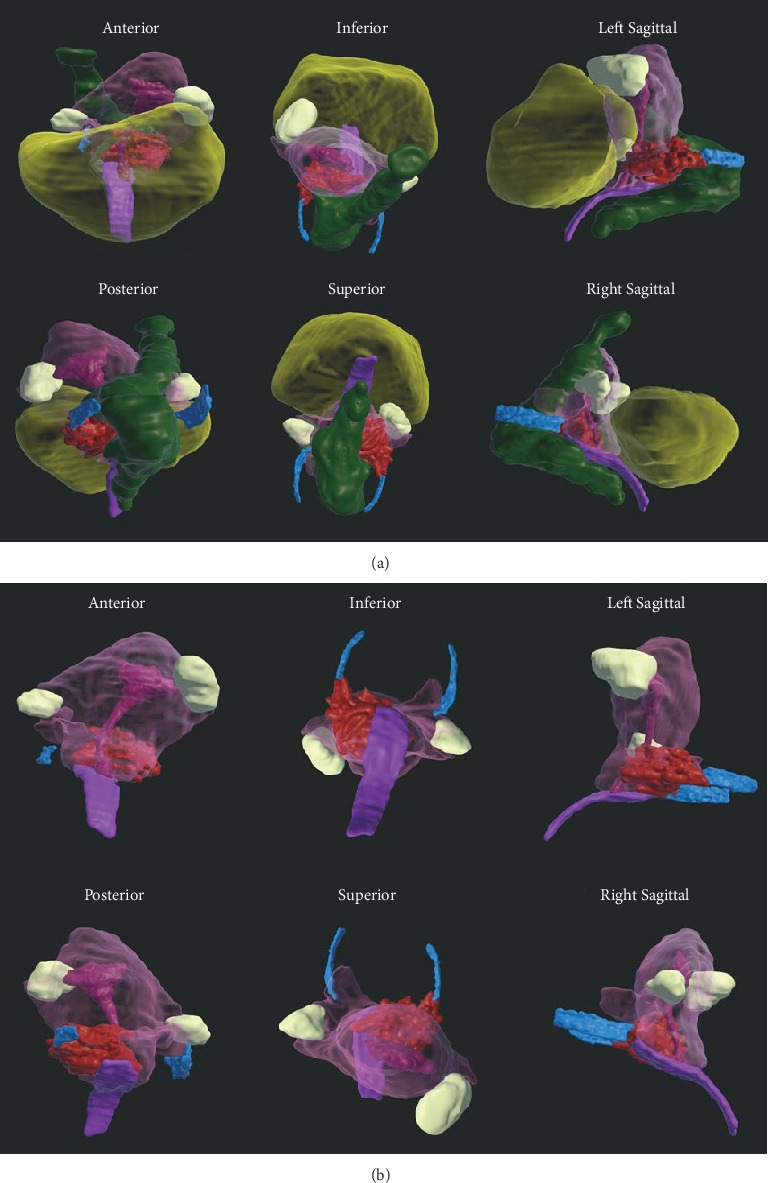
(a, b) 3D VR model viewed from the anterior–posterior, inferior–superior, and sagittal positions. The structures of interest are as follows: uterus + cervix + L hydrosalpinx is translucent pink, the endometrium is opaque magenta, the vagina is purple, endometriosis nodule is red, the uterosacral ligaments are blue, the bladder is yellow, and the rectum is green.

VR reconstruction was done to aid in surgical planning. Deidentified T2- and T1-weighted MR images were exported in DICOM format and imported into Elucis (v1.8.0, RealizeMedical, Ottawa, Canada), an advanced medical imaging visualisation software, for segmentation in a 3D VR environment (Video S1). The anatomy of interest was segmented using a combination of signal thresholding and manual editing to render 3D models of individual structures, including the vagina, cervix, uterus, endometrium, right fallopian tube, uterosacral ligaments, bowel, and deep endometrial lesions (Figure 3a,b). The final model can be viewed by the user/physician using a VR headset. The 3D structures of interest were also exported as a single object file to be viewed on a computer monitor using standard 3D viewer software. This VR reconstruction located the left parametrial endometriosis. The involvement of the left ureter, rectum, and vaginal canal, along with the vaginal mucosa, was delineated. Performing a virtual surgery at this stage helped in the precise and complete excision of the disease, thus preventing injury to the left ureter and rectum.

**Figure 4:**
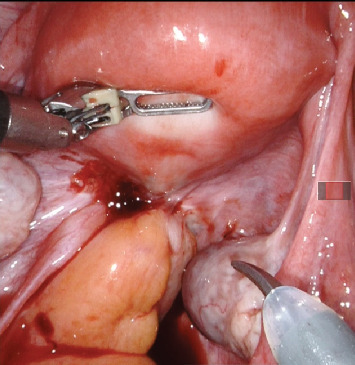
Initial appearance of the endometriosis involving the rectum, left ureter, and vaginal mucosa.

**Figure 5:**
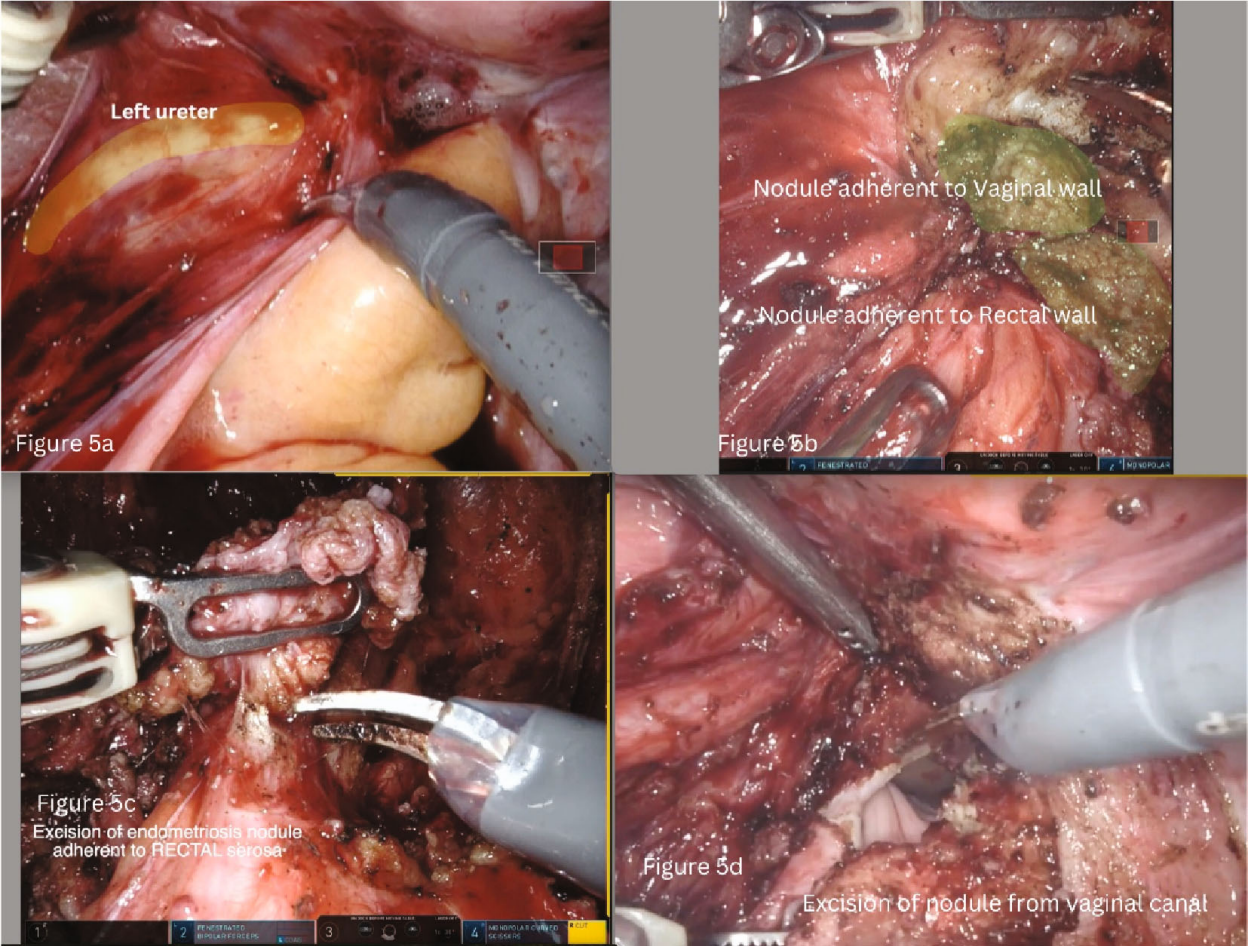
Dissection and excision of endometriotic nodule. (a) Position relative to the ureter, (b) nodule adherent to the vaginal wall, (c) excision of endometriosis nodules adherent to rectal serosa, and (d) excision of nodule from the vaginal canal.

**Figure 6:**
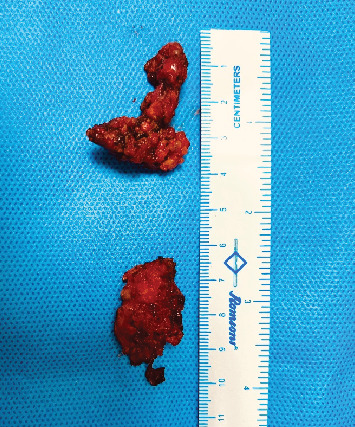
Excised endometriosis nodule in two pieces.

The procedure used a da Vinci Xi robot under general anaesthesia, modified lithotomy, and a Trendelenburg tilt of 22°. On initial evaluation, both adnexa and uterus were normal and devoid of endometriosis involvement (Figure 4). The left ovary was suspended to provide a clear surgical field. The left parametrial endometriosis nodule was identified, which involved the left ureter, vaginal muscularis, mucosa, and the rectal serosa. The left ureterolysis was performed, as the left ureter was externally encased with endometriosis and subsequent fibrosis. The left ureter was dissected and deflected laterally away from the line of excision. The dissection of the rectum began from the right side after identification of the right ureter at the pelvic brim and then proceeded to the left side. Both the medial (Okbayashi) pararectal spaces were dissected and identified. The dissection in the retrocervical area was then done. The nodule was split in half. One part was dissected along with the rectum attached to its serosa; this part of the left parametrial endometriosis nodule was then shaved off and removed. The other half of the left parametrial endometriosis nodule was attached to the vaginal canal. It was dissected by partial shaving off from the vaginal muscularis and partly by removing the full thickness of the vaginal canal along with the mucosa after opening the vaginal canal (Figures 5a, 5b, 5c, and 5d). The two parts of the left parametrial endometriosis nodule were retrieved vaginally, and the vaginal opening was closed with a polyglactin 1/0 suture (Figure 6).

### 2.1. Follow-Up at 18 Months

Her pain VAS score for dysmenorrhea has reduced to 2/10, and there was no dyspareunia (VAS 0/10). At the time of submission of this case, the patient had delivered at full term by cesarean section.

## 3. Discussion

Surgery is the definitive treatment for painful parametrial endometriosis. However, presurgical planning is the cornerstone for the precise excision of extensive endometriosis lesions. Adopting modern VR technology can advance this planning, in addition to traditional imaging (USG or MRI) [7]. VR technology enables the surgeon to become immersed in a computer-generated or anatomical depiction of both normal and abnormal anatomy. Being interactive, the surgeon can utilise this technology to find the anatomy and extent of the pathology and a virtual surgery (Video S1) before performing the live case. This is not possible with still images of MRI.

From a technical perspective, for cases evaluated with an MRI using T2-weighted sequencing as part of their standard preoperative planning, this VR and 3D modelling approach is most appropriate and can be offered. This aims to circumvent ethical considerations related to the aforementioned standard procedures. The VR technology is suitable for complex gynecologic conditions, such as deep and/or ovarian endometriosis, where multiorgan involvement is present. An uncomplicated unilateral small endometrioma or superficial endometriosis that does not appear on MRI may not be among the ideal cases present.

In our case, we have shown how an advanced visualisation technique like VR images and 3D models can provide details of the disease pattern. This allowed the robotic surgeon to plan their detailed surgical approach (two-step excision) and perform the surgery efficiently and effectively, achieving an optimal surgical outcome and minimal surgical complications. Similarly, in a series by Checcucci et al., all the surgeons decided to follow the suggested clamping strategy for partial nephrectomy, and the virtual model and real anatomy were a perfect match [8].

Aluwee et al. conducted a prospective randomised clinical trial for VR use in the presurgical planning of skull base tumour resection. VR technology was in localising lesions and designing patient-specific trajectories. This preplanning helped to reduce surgical time significantly. This resulted in fewer complications due to cerebrovascular injury and reduced postoperative stay [6].

Stadie et al. discussed the role of the Dextroscope as a workspace that allows for the display of 3D graphics stereoscopically to develop surgical approaches [9]. They reported that 93%, 83%, and 75% of surgeons felt that the device improved preoperative spatial understanding, craniotomy planning, and intraoperative spatial orientation and confidence, respectively.

Checcucci et al. published a series of nine cases of partial nephrectomy where metaverse preoperative clinical case discussion was used. The surgeons and moderator's digital avatars participated in the same virtual room, even though they were physically located in different hospitals, and discussed the surgical strategy [8].

Challenges with implementing 3D VR in a clinical setting are the cost of hardware and software as well as personnel training. The cost of hardware (computer and VR equipment) could be worldwide about $5000. However, one can acquire this formation for $2500 in India. The yearly licensing of the software can be expensive, up to $20,000 (but it is comparable to other 3D medical image postprocessing software). Approximately 2 h of training are needed to become acquainted with the software, as it is user-friendly. A longer learning curve is required in reading DICOM MRI images if one has no prior experience—the importance of a collaborative relationship with radiology. Such models are only as good as the images acquired with radiology colleagues to ensure appropriate modalities and sequences are being acquired for structures of interest. As for this case being discussed in this paper, the case was managed at Apollo Hospital, Hyderabad, India, and with collaboration at the Ottawa Hospital Research Institute, Canada, where the VR model was developed.

Recently, robotic surgery is gaining acceptance for the surgical management of endometriosis. Robotic surgery for endometriosis has certain potential benefits over traditional laparoscopy, especially in complex cases, and is deemed feasible and safe.

Robotic surgery can help to overcome the limitations of conventional laparoscopy.

However, studies show comparable outcomes between robotic and laparoscopic surgery regarding complication rates, pain relief, and quality of life improvements.

Due to the limited and heterogeneous nature of existing studies, long-term outcomes such as sustained pain relief, fertility rates, and quality of life are not well documented. This treatment can particularly benefit obese patients, those with a prior surgical history, and individuals with complex combinations of pelvic pathologies. It is especially advantageous for endometriosis involving the colorectal region, diaphragm, urinary tract, parametrium, and sacral plexus. Further well-designed studies are needed to clarify its role in managing endometriosis [10].

However, both robotic surgery and VR are new technologies, and this concept, when incorporated into clinical practice, has some drawbacks and difficulties. First, we must address the availability of such technology everywhere and the cost involved. In a country of a billion people (India), technology is reaching all corners quickly, and with an increased volume of use, the cost will ultimately come down. Secondly, the privacy and security of personal health records, consent, and data ownership must also be addressed. Few papers address legal implications and the use of VR technology in medicine. There are issues of registration, licensing, and practice across borders. Doctors and patients from different jurisdictions may use it, so it is essential to determine the legal framework in their geographic location. The question that needs recommendation is—Should doctors be licensed to offer such services? As these data are highly sensitive, privacy, confidentiality, and data protection are paramount. If the VR platform belongs to a third party, they may collect data. Effective regulations need to be considered in such a situation.

## 4. Conclusion

Advanced VR imaging modalities, in addition to ultrasound and MRI, can help diagnose and manage deep endometriosis. It can improve the surgeon's understanding of the specific anatomy, visualise the disease, and improve clinical outcomes. Multicentre collaboration can help realise the potential amalgamation of surgery and VR technology.

## Data Availability

The authors confirm that the data supporting the findings of this study are available within the article and its supporting information.

## Supporting Information

Supporting Information Additional supporting information can be found online in the Supporting Information section. Video S1: Elucis (v 1.8.0, RealizeMedical, Canada) virtual reality (VR) platform demonstrating medical image segmentation, design, and visualisation of endometriotic nodule involving the left uterosacral ligament, rectum, cervix, and vagina.
